# High-Temperature Stress Effect on the Red Cusk-Eel (*Geypterus chilensis*) Liver: Transcriptional Modulation and Oxidative Stress Damage

**DOI:** 10.3390/biology11070990

**Published:** 2022-06-29

**Authors:** Phillip Dettleff, Rodrigo Zuloaga, Marcia Fuentes, Pamela Gonzalez, Jorge Aedo, Juan Manuel Estrada, Alfredo Molina, Juan Antonio Valdés

**Affiliations:** 1 Escuela de Medicina Veterinaria, Facultad de Agronomía e Ingeniería Forestal, Facultad de Ciencias Biológicas y Facultad de Medicina, Pontificia Universidad Católica de Chile, Santiago 7820436, Chile; 2 Facultad de Ciencias de la Vida, Universidad Andrés Bello, Santiago 8370186, Chile; rodrigo.zuloaga.r@gmail.com (R.Z.); fvmarcia@gmail.com (M.F.); pamela.gonzalez.tr@gmail.com (P.G.); jor.aedo.a@gmail.com (J.A.); amolina@unab.cl (A.M.); 3 Interdisciplinary Center for Aquaculture Research (INCAR), Concepción 4030000, Chile; 4 Centro de Investigación Marina Quintay (CIMARQ), Universidad Andrés Bello, Quintay 2340000, Chile; mestrada@unab.cl

**Keywords:** red cusk-eel, thermal stress, RNA-seq, liver transcriptome, oxidative damage, protein folding, hepatic enzymes

## Abstract

**Simple Summary:**

The red cusk-eel (*Genypterus chilensis*) is a native Chilean species important for aquaculture diversification in Chile. The effect of high-temperature stress on the liver, a key organ for fish metabolism, is unknown. In this study we determined for the first time the effects of high-temperature stress on the liver of red cusk-eel. The results showed that high-temperature stress increased hepatic enzyme activity in the plasma of stressed fish. Additionally, this stressor generated oxidative damage in liver, and generated a transcriptional response with 1239 down-regulated and 1339 up-regulated transcripts associated with several processes, including unfolded protein response, heat shock response and oxidative stress, among others. Together, these results indicate that high-temperature stress generates a relevant impact on liver, with should be considered for the aquaculture and fisheries industry of this species under a climate change scenario.

**Abstract:**

Environmental stressors, such as temperature, are relevant factors that could generate a negative effect on several tissues in fish. A key fish species for Chilean aquaculture diversification is the red cusk-eel (*Genypterus chilensis*), a native fish for which knowledge on environmental stressors effects is limited. This study evaluated the effects of high-temperature stress on the liver of red cusk-eel in control (14 °C) and high-temperature (19 °C) groups using multiple approaches: determination of plasmatic hepatic enzymes (ALT, AST, and AP), oxidative damage evaluation (AP sites, lipid peroxidation, and carbonylated proteins), and RNA-seq analysis. High-temperature stress generated a significant increase in hepatic enzyme activity in plasma. In the liver, a transcriptional regulation was observed, with 1239 down-regulated and 1339 up-regulated transcripts. Additionally, high-temperature stress generated oxidative stress in the liver, with oxidative damage and transcriptional modulation of the antioxidant response. Furthermore, an unfolded protein response was observed, with several pathways enriched, as well as a heat shock response, with several heat shock proteins up regulated, suggesting candidate biomarkers (i.e., *serpinh1*) for thermal stress evaluation in this species. The present study shows that high-temperature stress generated a major effect on the liver of red cusk-eel, knowledge to consider for the aquaculture and fisheries of this species.

## 1. Introduction

Environmental factors are important for the physiology of fish, particularly those associated with water conditions. Among these, temperature, pH, and dissolved oxygen (DO) are crucial for the homeostasis of the fish in marine environments, and changes in these factors could lead to generating a stressful status for the animal [1]. It has been reported that an increase in water temperature could lead to stress and negative effects on marine fish, including salmonids [2,3], catfish [4], Atlantic cod [5], and gilthead seabream [6]. Understanding the effects of water temperature on fish is crucial in the actual scenario of climate change, considering the average rising sea temperature per decade [7]. Additionally, the effect of climate change on sea temperatures is expected to influence relevant phenomena of the Pacific coast, such as El Niño–Southern Oscillation (ENSO), increasing their intensity and frequency [8], which is relevant for marine species of the South Pacific coast, considering the increase in water temperatures associated with this phenomena. Therefore, it is important to understand the effect of an increase in temperature and how it affects the stress response in marine fish with aquaculture potential.

Chile is a relevant country in seafood production, with an important fishery and aquaculture industry. Additionally, the Chilean aquaculture sector is recognized for its important salmon and mussel industries [9]. However, in the last decade, an important effort has been made by the public private association to diversify Chilean aquaculture with native fish of commercial value. One of these species is the red cusk-eel (*Genypterus chilensis*), part of the *Genypterus* genus, endemic to the South Pacific coast, an economically relevant fish for fisheries and, recently, part of the Chilean aquaculture diversification program [10,11], with recent elucidation of the complete production cycle [12]. The red cusk-eel is a demersal fish with a carnivorous diet; it lives on rocky bottoms [13] and is characterized reproductively as a multiple spawner [11]. The high value of its flesh makes this species an attractive product; however, tons of fisheries have presented variable levels, with a decreasing tendency in the last decade [14]. In this sense, it is important to understand how environmental factors could affect this species and the mechanisms involved.

Stress in fish can be characterized as an adaptative response to danger, which generates physiological changes to prepare the fish to respond and survive threats. This stress response in fish is mediated by the hypothalamic–pituitary–interrenal (HPI) axis though several key chemical mediators, including corticotropin releasing factor, adrenocorticotropic hormone, α-melanocyte-stimulating hormone, adrenaline, and cortisol, a key hormone that increases in plasma under stress [15]. If the stressor is maintained for prolongated periods, a chronic stress status is generated in the fish, leading to several negative physiological effects, including decreased growth, reproductive problems, behavior modifications, and immune response [16,17]. Moreover, at a cellular level, the stress can lead to an increase in reactive oxygen species (ROS), which could lead to an oxidative stress status, as previously observed for several teleost fish species [18,19,20,21,22], an effect also observed for red cusk-eel in response to several stressors [23,24,25]. Red cusk-eel has shown low tolerance to intensive farming stressors, with limited information related to the stress response capacity in this species [13]. However, the specific stress response varies according to species, as well as the effect on each tissue. Our previous studies on *G. chilensis* showed a variable tissue response under handling stress, with altered metabolic status in the liver, a modulation of the immune response in the head kidney, and an induction of atrophy in skeletal muscle through coding and noncoding regulation [26]. Additionally, it has been observed that thermal stress could induce muscle atrophy in this species [24], as well as oxidative damage in eggs, with a minor effect on the ovary [23]. One of the most important organs for fish metabolism is the liver, which is directly involved in stress response by metabolizing and liberating stored energy to respond to stress [17]. Nevertheless, the liver response to thermal stress in *Genypterus* species has not been previously studied, nor has the impact of this stressor on the oxidative status of this tissue. It is important to consider that sea temperatures will increase through sea heat waves due to the effect of ENSO under a climate change scenario, which will affect the Chilean coast associated with the geographic range of *G. chilensis*. Therefore, the objective of this study was to evaluate the effect of high-temperature stress on the liver of *G. chilensis* in terms of the transcriptomic and oxidative stress status to determine the negative impact of this type of stressor on liver.

## 2. Materials and Methods

### 2.1. Ethics Statement

All procedures with the red cusk-eel individuals and all scientific activities adhered to animal welfare procedures and were approved by the bioethical committees of the Universidad Andres Bello (007/2018) and the National Commission for Scientific and Technological Research (CONICYT) of the Chilean government.

### 2.2. Fish Sampling and Experimental Design

In this study, we used reproductively immature red cusk-eel juveniles (*G. chilensis*) of 12 months of age (average weight of 665 ± 52.7 g; average length of 60 ± 4.8 cm), collected from the Centro de Investigación Marina de Quintay (CIMARQ), maintained under natural photoperiod conditions (L:D 12:12), and controlled temperature (14 ± 1 °C), and fed daily with commercial pellet food. Fish were separated into control and stress groups, with the stress group subjected to a standardized thermal stress protocol proven to generate stress in red cusk-eel [24]. Briefly, this protocol consists of increasing the temperature over 24 h at a rate of 1 °C in 5 h. This protocol maintains the thermal stress temperature (19 ± 1 °C) for 5 days. This high temperature protocol was selected considering heat waves observed in the summer season on the Chilean coast in recent years [27]. The control group was maintained at the control temperature (14 ± 1 °C) during the assay. At the end of the experiment, six individuals per group (two tanks per group, with three animals sampled per tank, total of N = 12 sampled fish) were netted and sampled. Blood samples were collected via caudal puncture using heparinized tubes, immediately centrifuged at 5000× *g* for 10 min for serum obtention and stored at −80 °C until analysis. After blood sampling, fish were euthanized (overdose of anesthetic 3-aminobenzoic acid ethyl ester, 300 mg/L). Fish livers were collected and stored for RNA extraction in RNAsave solution (Biological Industries, Cromwell, CT, USA) or immediately frozen in liquid nitrogen and stored at −80 °C until analysis for oxidative damage evaluation.

### 2.3. AST, ALT and AP Evaluation

The plasmatic activities of aspartate aminotransferase (AST), alanine aminotransferase (ALT), and alkaline phosphatase (AP) were determined using commercially available kits from Valtek (Santiago, Chile) following the manufacturer’s instruction. Briefly, these kits determined the enzymatic activity (IU/L) via the generation of colorimetric products from glutamate (colorimetric product: 450 nm), pyruvate (535 nm), and p-nitrophenol (405 nm) for AST, ALT and AP activity, respectively.

### 2.4. Oxidative Stress Assays in Liver

To determine the oxidative damage in the liver of red cusk-eel in response to high-temperature stress, DNA oxidative damage, protein carbonylation, and lipid peroxidation were evaluated using commercially available kits. The DNA oxidative damage assay was performed with genomic DNA (gDNA) purified from 25 mg of the liver using the DNAzol reagent (Invitrogen, Carlsbad, CA, USA) following the manufacturer’s protocol and quantified with the Epoch Spectrophotometer System (BioTek, Winooski, VT, USA). Then, the apurinic/apyrimidinic sites (AP sites) were determined in the gDNA using the OxiSelect Oxidative DNA Damage Quantification Kit (Cell Biolabs, CA, USA) following the manufacturer’s protocol. The protein carbonylation assay was performed using total protein extracted from 100 mg of the liver in 1 mL of lysis buffer containing 50 mM Tris-HCl pH 7.4, 150 mM NaCl, 1 mM EDTA, and 1% NP-40, solubilized at 4 °C after 12,000× *g* centrifugation. Proteins were quantified using the Pierce BCA Protein Assay Kit (Thermo Scientific, Batavia, IL, USA). Then, carbonylated protein content was quantified using the OxiSelect Protein Carbonyl Spectrophotometric Assay (Cell Biolabs, San Diego, CA, USA) following the manufacturer’s protocol. The lipid peroxidation determination in the liver was performed using the OxiSelect HNE Adduct Competitive ELISA Kit (Cell Biolabs, San Diego, CA, USA), following the manufacturer’s protocol. This kit determines lipid peroxidation through the quantification of hydroxynonenal (HNE) protein adducts in the extracted proteins of the liver.

### 2.5. Liver RNA Extraction, Library Preparation and Illumina Sequencing

Total RNA was extracted from the liver stored in RNAsave solution (Biological Industries, Cromwell, CT, USA) using the TRIzol^®^ reagent (Invitrogen, Carlsbad, CA, USA) protocol. Total RNA was quantified by fluorometry with the Qubit^®^ RNA quantitation assay (Invitrogen, Carlsbad, CA, USA) and purity was determined according to 260/280 ratio using the Epoch Spectrophotometer System (BioTek, Winooski, VT, USA). The RNA integrity was measured according to RNA Quality Measurement Number (RQN) through a Fragment Analyzer with the Standard Sensitivity RNA Analysis kit (Advanced Analytical Technologies, Fiorenzuola, Italy), selecting samples with RQN ≥ 8. The cDNA libraries construction were performed with 1 µg of total RNA per sample using the Illumina^®^ TruSeq RNA Sample Prep Kit v2 (Illumina^®^, San Diego, CA, USA), following the manufacturer’s protocol. The sizes of the mRNA libraries were determined through a Fragment Analyzer using the NGS Fragment Analysis kit (Advanced Analytical Technologies) and quantified by qPCR using the Kapa Library Quantification kit (Roche, Little Falls, NJ, USA). Paired-end sequencing (2 × 100 bp) was performed on a Hiseq 2500 (Illumina^®^) platform in Macrogen Inc. (Seul, South Korea).

### 2.6. Reads Filtering, Differential Expression, and GO Enrichment Analysis

The raw reads obtained from Illumina sequencing were trimmed to remove the remaining Illumina adapter, low-quality sequences, and short sequences (<50 bp), using the CLC Genomics Workbench v.7.0.3 software. The filtered reads were mapped to a *G. chilensis* reference transcriptome previously annotated by our group ([28], NCBI accession number SRS614525) using the CLC Genomics Workbench v.7.0.3 software with the following parameters: mismatch cost = 2, insertion cost = 3, deletion cost = 3, length fraction = 0.8, and a similarity fraction = 0.8. The expression values were used for clustering and heatmap chart generation with R. Differential expression analysis was performed with the R package DESeq2 (version 1.2.10) [29] to determine differentially expressed transcripts in the liver between the control and stress groups. Transcripts presenting an adjusted *p*-value of <0.05 and an absolute log2 fold change of >1 were considered as differentially expressed between the groups. Enrichment analysis was performed on the list of differentially expressed transcripts and GO terms to determine the overrepresented processes in response to high-temperature stress in the liver, considering up and down-expressed transcripts. This analysis was performed using the enrichment analysis tool implemented in the Blast2GO software [30]. Additionally, the Kyoto Encyclopedia of Genes and Genomes (KEGG) metabolic pathway database was used to build the represented pathways through the KEGG Automatic Annotation Server (KAAS) [31] using the KEGG Orthology (KO) identifiers of the differentially expressed list.

### 2.7. RNA-seq Validation by qPCR

Total RNA previously extracted was DNAse-treated to remove residual gDNA, and 1 μg of RNA was reverse transcribed into cDNA using the QuantiTect^®^ Reverse Transcription kit (Qiagen, Germantown, MD, USA), following the manufacturer’s protocol. A total of thirteen differentially expressed transcripts were selected for qPCR validation, corresponding to: *hsp60*, *hsp70*, *gpx7*, *ddit4*, *leptin*, *msh2*, *msh3*, *c1ql1*, *ccl20*, *atg12*, *atg4b*, *casp3* and *c3*. Primers were designed with Primer 3 software v0.4.0 (http://frodo.wi.mit.edu/primer3/, accessed on 6 May 2021) using the reference transcriptome previously described. The primer sequences, amplicon size, Tm, and efficiency are presented in Table 1. The qPCR was performed in a Stratagene MX3000P qPCR system (Stratagene, La Jolla, CA, USA). All qPCR assays were performed in triplicates, using no-template and no-RT controls, in compliance with the MIQE guidelines [32]. The qPCR reaction mixture contained 7.5 μL of 2× Brilliant^®^ II SYBR^®^ Green master mix (Agilent Technologies, CA, USA), 100 ng of cDNA per reaction and 200 nM of each primer in a 15 μL final volume. Thermal cycling conditions were an initial activation of 2 min at 95 °C, followed by 40 cycles of 30 s at 95 °C, 30 s at 62 °C, and 30 s at 72 °C. A melt curve analysis was performed to confirm a single qPCR product and a standard curve using two-fold series dilutions was used to estimate the efficiency of each primer set. The expression of target genes was normalized using the geometric means of two reference genes (*actb* and *taf12*) previously validated for red cusk-eel in the liver [26] and following the methodology described by [33].

### 2.8. Statistical Analysis

Significant differences between means of the control and stress groups for DNA damage (AP sites), enzymatic activity (AST, ALT, and AP) and differential expression of the qPCR validated genes were determined using a *t*-test with a significance threshold of *p* < 0.05. All statistical analyses were performed using GraphPad Prism, v.5.00 (GraphPad Software, San Diego, CA, USA).

## 3. Results

### 3.1. Hepatic Enzyme Activity and Oxidative Stress Response to High-Temperature Stress

**Figure 1:**
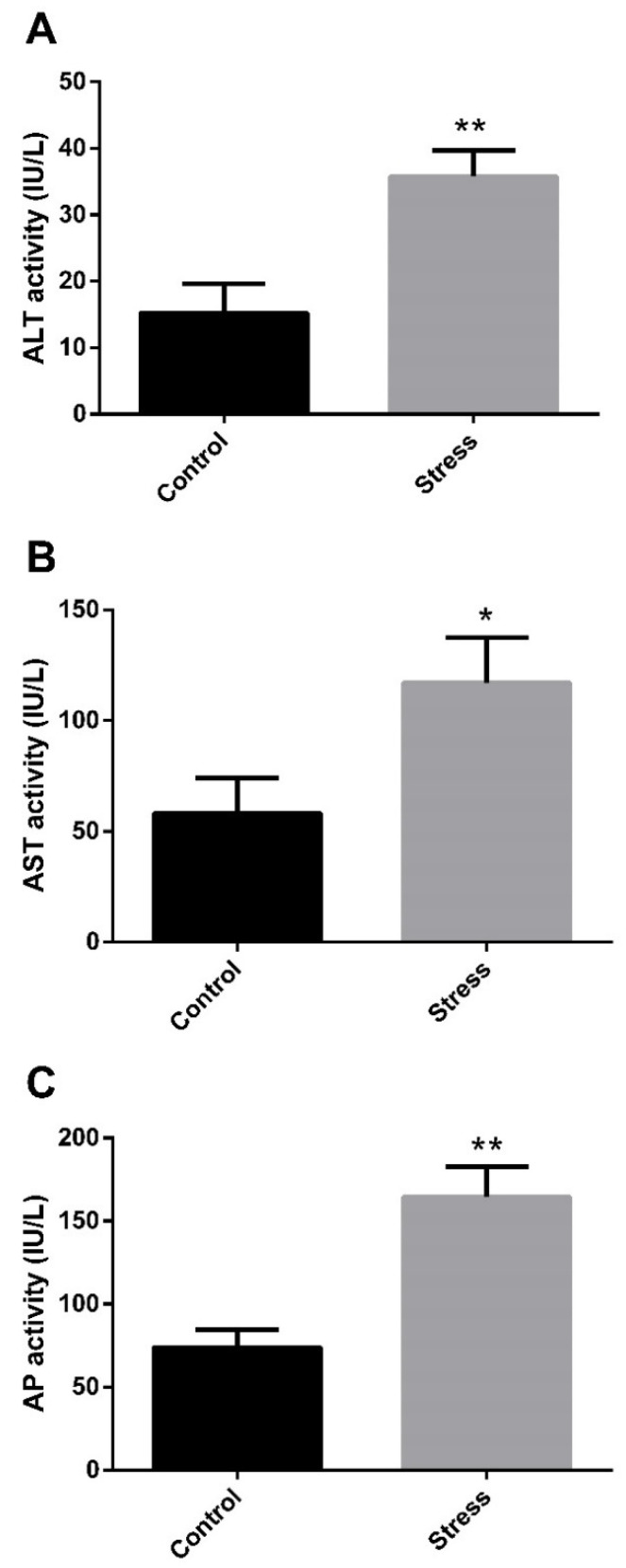
Plasmatic activity of alanine aminotransferase (ALT), aspartate aminotransferase (AST), and alkaline phosphatase (AP) in control and high-temperature stress groups of *G. chilensis.* Levels of ALT (**A**), AST (**B**), and AP (**C**) activity are expressed in IU/L. Bars represent the mean ± SEM. Significant differences between the control and stress groups are indicated by asterisks; * (*p* < 0.05) and ** (*p* < 0.01).

**Figure 2:**
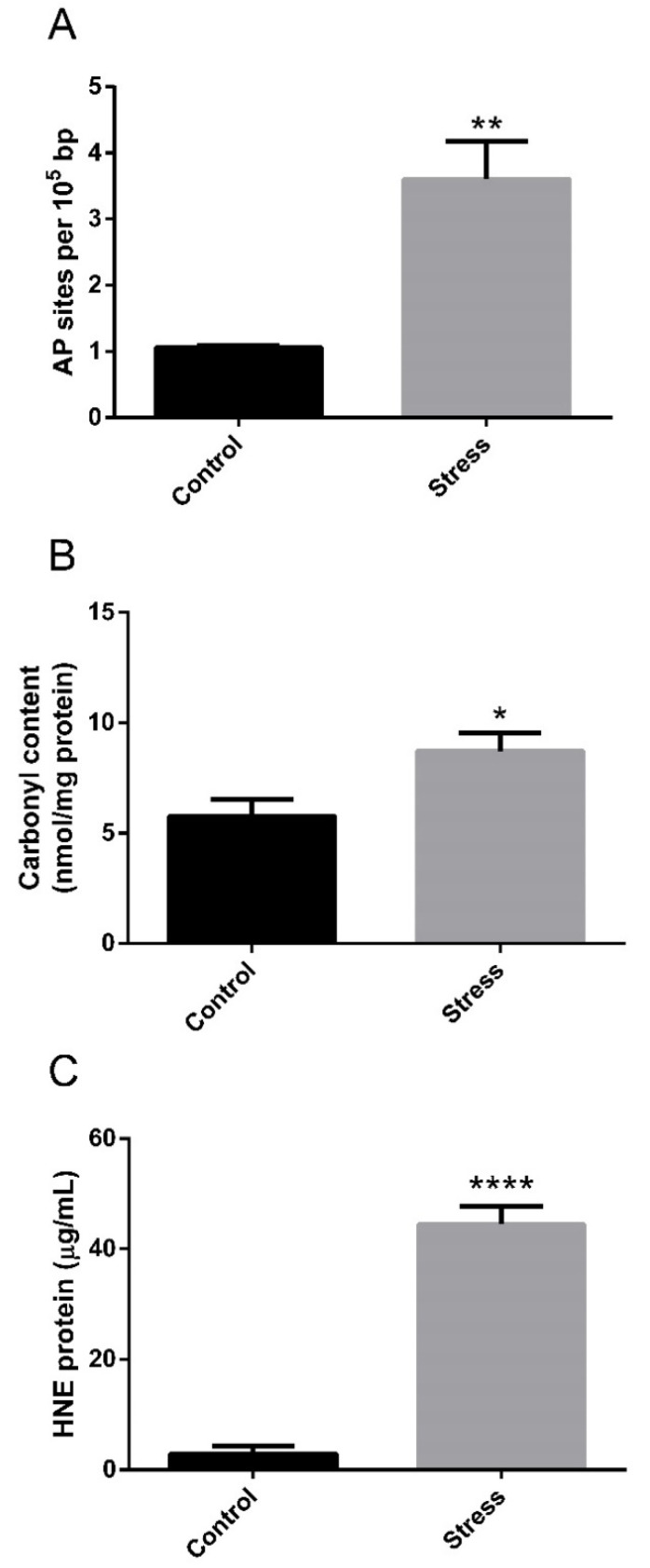
Evaluation of oxidative damage in the liver of *G. chilensis* in control and high-temperature stress groups. Level of DNA damage in terms of AP sites (**A**), protein carbonylation (**B**), and lipid peroxidation in terms of HNE adducts (**C**) in control and stress groups. Bars represent the mean ± SEM. Significant differences between the control and stress groups are indicated by asterisks; * (*p* < 0.05), ** (*p* < 0.01) and **** (*p* < 0.0001).

We previously reported that thermal stress for 5 days significantly increased plasmatic levels of cortisol and glucose in *G. chilensis* [24]. To understand how thermal stress affects the metabolism in this species, we studied the effect of high temperature on the liver. To evaluate the effect on the hepatic function of this type of stressor, we measured plasmatic markers of liver damage, i.e., ALT, AST, and AP enzymatic activity. High-temperature stress significantly increased the plasmatic activity of ALT (Figure 1A), AST (Figure 1B) and AP (Figure 1C) in the stress group, evidencing altered hepatic function in the liver of stressed fish. To determine the oxidative damage in the liver generated by high-temperature stress, the DNA oxidative damage, protein carbonylation and lipid peroxidation were determined in the liver. High temperatures generated DNA damage, evidenced by the significant increase in apurinic/apyrimidinic sites in the stressed group (Figure 2A). Oxidative damage was also observed in lipid and proteins, determined by a significant increase in protein carbonylation (Figure 2B) and lipid peroxidation (Figure 2C) in response to high temperature in the stress group.

### 3.2. Differentially Expressed Transcripts in Hepatic Response to High-Temperature Stress

**Figure 3:**
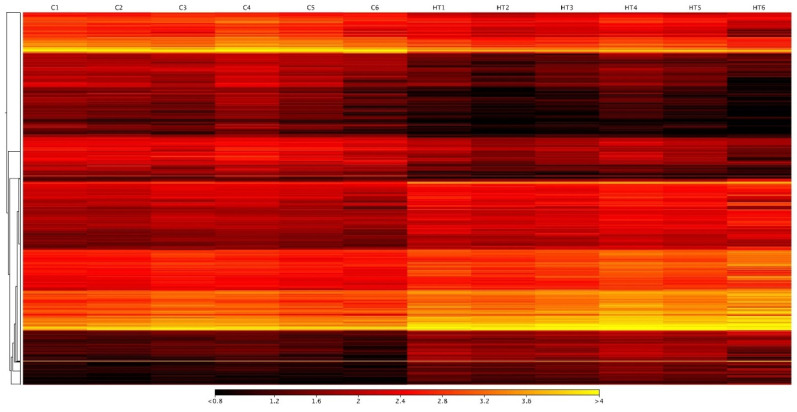
Differentially expressed transcripts in response to high-temperature stress in *G. chilensis.* The heatmap presents the differentially expressed transcripts between control (C) and stress groups (HT). The numbers 1 to 6 indicate the sample library of control and stress fish.

To understand the complexity of the stress response in the liver of *G. chilensis,* we performed RNA-seq analysis on the liver of each experimental group. The sequencing generated a total of 754,678,455 paired-end reads, with an average of 58,052,189 raw paired-end reads per library. The raw data are available from NCBI under BioProject PRJNA835467 with BioSamples accession number SAMN28102858, SAMN28102859, SAMN28102860, SAMN28102861, SAMN28102862, SAMN28102863, SAMN28102864, SAMN28102865, SAMN28102866, and SAMN28102867. After trimming by quality, adapters, and size, we obtained an average of 58,018,122 high-quality filtered paired-end reads per library (Table 2). These reads were mapped to the *G. chilensis* reference transcriptome (NCBI accession number SRS614525), obtaining an average of 85.2% mapped reads (Table 2). Expression values were used for normalization and differential expression analysis with the R package DESeq2 (version 1.2.10) [29], obtaining a total of 2578 differentially expressed transcripts between control and stressed groups. Of these transcripts, 1239 were down-regulated (Table S1) and 1339 were up-regulated (Table S2) in the stressed group (Figure 3 and Figure S1), evidencing five clusters of transcripts with different patterns of expression for the control and thermal stress groups (Figure S2).

### 3.3. GO Enrichment and Pathway Analysis in the Liver

The differentially expressed transcripts were used for an enrichment analysis using GO terms to determine the overrepresented processes in response to high-temperature stress in the up- and down-regulated transcripts of the liver. The enrichment analysis showed 38 enriched processes for up-regulated transcripts (Table 3), including 18 biological processes (BP), 16 cellular components (CC), and 4 molecular functions (MF). Several of the enriched terms were related to protein metabolism, including protein folding (GO:0006457), protein transport (GO:0015031), protein localization (GO:0008104), protein retention in ER lumen (GO:0006621), and unfolded protein binding (GO:0051082). Additionally, the BP term response to heat (GO:0009408) was also enriched in the up-regulated transcripts in the high-temperature stress group. No enriched GO terms were identified in the down-regulated transcripts (FDR < 0.05).

**Figure 4:**
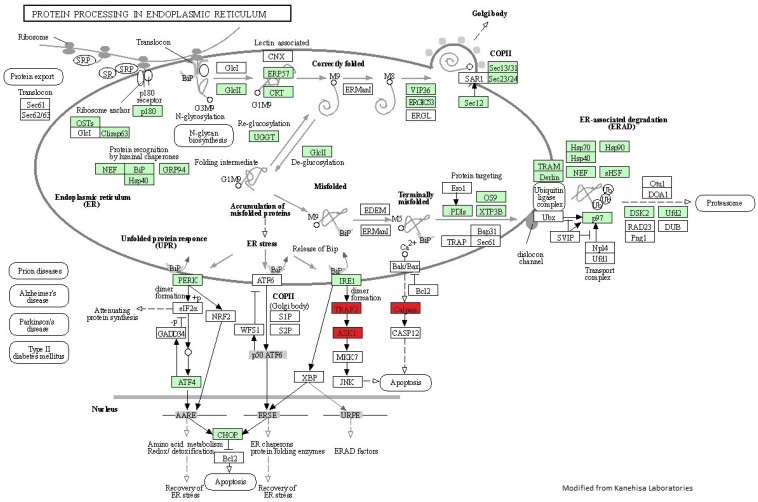
Illustration of transcriptional changes in liver of *G. chilensis* in response to high-temperature stress using modified KEGG pathway map of protein processing in the endoplasmic reticulum. The red rectangles indicate down-regulated transcripts, while green rectangles indicates up-regulated transcripts between the control and high-temperature groups.

In the KEGG pathway analysis, we identified several pathways represented in the down-regulated and up-regulated transcripts, including apoptosis, cell cycle, MAPK signaling pathway, autophagy, protein processing in endoplasmic reticulum, and ubiquitin-mediated proteolysis. Among these pathways, protein processing in endoplasmic reticulum was one of the most represented on the differentially expressed transcripts (Figure 4), with 31 up-regulated and three down-regulated transcripts represented in this pathway, including several heat shock proteins; heat shock protein 40 (*hsp40*, also known as *dnaJ homolog subfamily B member 1*), heat shock protein 70 (*hsp70*) and heat shock protein 90 (*hsp90*).

### 3.4. qPCR Validation of Differentially Expressed Genes

**Figure 5:**
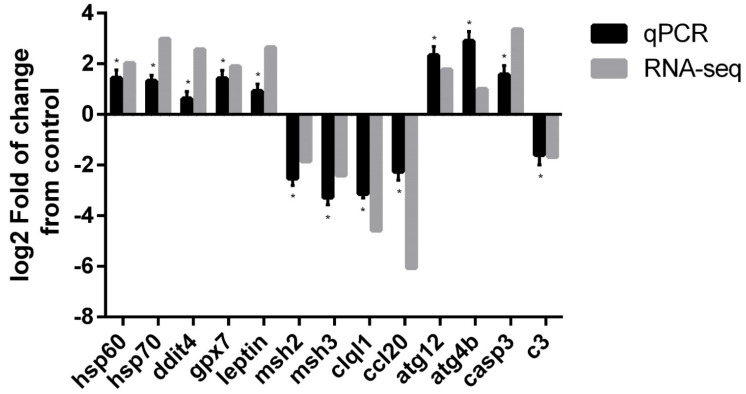
qPCR validation of selected differentially expressed transcripts in the liver of *G. chilensis* in response to high-temperature stress. The transcript expression levels were normalized with the geometric means of *actb* and *taf12*. The differential expression levels according to qPCR (black bars) and RNA-seq (gray bars) for these selected genes are expressed as log_2_ fold changes. The log_2_FC represents the expression change in the stress group compared with the control group. Results are expressed as the mean ± standard error. Significant differences in the validated qPCR data between control and stress groups are indicated by asterisks in the log2 Fold qPCR bars; (* *p*-value < 0.05).

The validation of the RNA-seq results was performed by selecting 13 differentially expressed transcripts between the control and stressed groups, including heat shock protein 60 (*hsp60*), hsp70, DNA damage-inducible transcript 4 (*ddit4*), glutathione peroxidase 7-like (*gpx7*), leptin, DNA mismatch repair Msh2-like (*msh2*), DNA mismatch repair Msh3-like (*msh3*), complement C1q 2-like (*clql1*), C-C motif chemokine 20-like (*ccl20*), ubiquitin ATG12-like (*atg12*), cysteine protease ATG4B-like (*atg4b*), caspase 3 (*casp3*) and complement C3-like (*c3*) (Table 1). All of the selected differentially expressed transcripts on RNA-seq were validated by qPCR, confirming the results of the RNA-seq analysis (Figure 5), with a Pearson’s correlation coefficient of r = 0.847, confirming the significant differential expression of all tested transcripts by qPCR between the control and high-temperature group (Figure S3).

## 4. Discussion

Stress in fish is a relevant issue for aquaculture species and native populations of fish [15]. The environmental stress factor associated with water parameters represents a key issue in fish, especially considering the variations in the environment in the short- and long-term associated with global warming, climate change, ENSO, and pollution of the oceans, which could lead to modification of sea temperature, pH, and DO level, as well as increased levels of pollutants, including microplastics and toxic compounds, which could generate stress in teleost fish [7,8,34,35,36]. These environmental stressors could also affect the red cusk-eel, a relevant species for Chilean fisheries and aquaculture diversification. However, studies aimed at understanding the effect of stressors in *Genypterus* species are limited [25,26,37,38], with no information about the impact of thermal stress on the liver for *Genypterus* species. In this sense, thermal stress has been previously studied in other tissues of *Genypterus* species, including the skeletal muscle, ovary, and post-ovulatory eggs [23,24]. Therefore, to understand the effect of high-temperature stress at the hepatic level, we evaluated the hepatic enzymes, oxidative stress response, and transcriptional regulation in red cusk-eel.

### 4.1. High-Temperature Effect on Hepatic Enzymes

In a previous study, we determined that high temperature (19 °C) could generate a stress response in red cusk-eel with an increase in the plasmatic level of cortisol and glucose [24]. In the present study, we determined that a high temperature increased the plasmatic activity of ALT, AST, and AP enzymes, evidencing the effect at the hepatic level. This effect was previously observed in other teleost fish under stress conditions, including the plasmatic activity of ALT and AST under high densities and metal pollution in rohu (*Labeo rohita*) [39,40], metal toxicity and pesticides in Nile tilapia (*Oreochromis niloticus*) [41,42] and spotted snakehead (*Channa punctatus*) [43], and elevated ALT, AST and AP under handling stress in red cusk-eel [25]. In terms of high-temperature stress, different results have been observed, with an increase in the plasmatic activity of AST and ALT in pufferfish (*Takifugu obscurus*) [44], which is consistent with our results for red cusk-eel, while no effect for AST and ALT was observed in Turbot (*Scophthalmus maximus*) [45] under high-temperature stress, evidencing that the hepatic response to thermal stress could vary according to fish species. The increased levels on ALT, AST an AP enzymes could be indicative of liver dysfunction, reflecting hepatocyte damage due to thermal stress, which is concordant with the results observed in mammals [46].

### 4.2. Oxidative Stress under High-Temperature Stress

Oxidative stress corresponds to a disturbance between the production of ROS, which can accumulate in cells, and the antioxidant defenses generated by the cellular systems to detoxify these ROS [47]. OS participate in different normal cellular functions, acting as a second messenger in signal transduction [48], but they can also generate cellular damage, including oxidative damage generating lipid peroxidation, as well as damage to proteins and DNA [49]. Environmental stress, including thermal stress, can lead to oxidative stress status in marine animals [3]. It was previously observed that thermal stress could modulate the oxidative stress status in several tissues of teleost fish [50,51]. One of the relevant organs in which oxidative status could be affected by thermal stress is the liver, as it was previously observed that thermal stress by low temperature could increase antioxidant enzymes in milkfish (*Chanos Chanos*) [52], as well as generate oxidative damage, leading to lipid peroxidation in Pacu (*Piaractus mesopotamicus*) under low-temperature stress [53,54]. High-temperature stress could also generate a relevant impact on the oxidative status of fish, with increased antioxidant enzyme activities, as observed for Senegalese sole (*Solea senegalensis*) [55]. Additionally, oxidative damage was observed in the liver of rohu (*Labeo rohita*), with lipid peroxidation and DNA fragmentation [56]. This is consistent with the findings of our study, evidencing that thermal stress generates an important oxidative effect on the liver of red cusk-eel. This response was also observed at the transcriptional level, with the up-regulation of GPx genes in several tissues of teleost fish under thermal stress, including black porgy (*Acanthopagrus schlegeli*) [57] and pufferfish [58], as well as in red cusk-eel eggs under thermal stress [23], where *gpx1* was increased, an effect not observed for this species in skeletal muscle [24]. Additionally, the effect of high-temperature stress on the liver related to DNA damage was also observed at the transcriptional level, with the up-regulation of genes involved in DNA mismatch repair (*msh2* and *msh3*), concordant with the previously reported effect of thermal stress on zebrafish (*Danio rerio*) [59] and American lobster [60]. However, the thermal stress response associated with oxidative stress in teleost fish could vary according to species and tissues, as observed in sheepshead minnow (*Cyprinodon variegatus*), where a limited effect on antioxidant enzymes and no lipid peroxidation were present [61], in contrast to the variable lipid peroxidation observed in Senegalese sole [55]. We previously observed in red cusk-eel that the impact of high-temperature stress on oxidative damage could vary according to tissue, with lipid peroxidation and DNA damage observed for skeletal muscle [24], but no oxidative damage was observed in the ovaries under high-temperature stress [23], showing that the liver is a sensitive organ under thermal stress in red cusk-eel under an ENSO temperature increase scenario.

### 4.3. High-Temperature Stress in Hepatic Protein Processing and Folding

Temperature can modulate several cellular processes through gene expression regulation [2]. Here, we used RNA-seq analysis to evaluate, for the first time, the effect of thermal stress on the liver of red cusk-eel, considering that no studies have previously evaluated the temperature effect on the liver in any species of the *Genypterus* genus. We observed a higher hepatic transcriptional response to high-temperature stress compared to other types of stressors, such as handling stress, previously observed for red cusk-eel (4.6 times the differentially expressed transcripts) [25], evidencing that a high temperature could have a higher transcriptional impact on the liver than others stressors in this species. At the cellular level, the function of endoplasmic reticulum is key to protein synthesis, folding, and exporting [62]. However, external processes such as thermal stress could generate alterations in homeostasis, affecting normal protein folding, which leads to endoplasmic reticulum (ER) stress. To alleviate this stress, the unfolded protein response molecular mechanism is activated in cells [63]. This ER stress and subsequent unfolded protein response is concordant with our results in the liver, where we observed enriched processes associated with this type of response in the up-regulated genes in the stress group, including protein folding, protein transport, protein localization, protein retention in ER lumen, and unfolded protein binding. In this sense, we found several genes associated with protein processing and folding pathways, including transcripts involved in: protein export, such as signal recognition particle 14 and 19 kDa (*srp14* and *srp19*) and signal peptidase complex subunit 2-like (*spcs2*) [64]; transcripts associated with protein processing in endoplasmic reticulum (Figure 4), with part of this pathway down-regulated, including TNF receptor-associated factor 2-like (*traf2*), and mainly an up-regulation of this pathway, including ubiquilin-4 (*ubqln4*), cytoskeleton-associated protein 4 (*ckap4*); ribosome-binding protein 1 (*rrbp1,* also known as *p180*); and glucosidase 2 subunit beta (*prkcsh*). Others include transcripts associated with the unfolded protein response, including eukaryotic translation initiation factor 2 alpha kinase 1 (*eik2ak1,* also known as *hri*), 3 (*eik2ak3*, also known as *perk*) and 5 (*eik2ak5*), cyclic AMP-dependent transcription factor ATF-4-like (*atf4*) and DNA damage-inducible transcript 3 -like (*ddit4,* also known as *chop*). The unfolded protein response could be initiated by EIK2AK3 kinase activation of eIF2α, leading to ribosome inhibition and attenuating protein synthesis [65]. Additionally, the ATF4 gene was activated, which would lead to DDIT4 gene regulation and an antioxidant response in cells [66], evidencing that thermal stress in the liver of red cusk-eel modulates the protein processing and generates an unfolded protein response that is not able to control the oxidative stress and damage in this tissue. This is concordant with the results observed in previous studies where unfolded protein response genes were activated under thermal stress in mammals [67,68] and fish, e.g., in the liver of Tambaqui (*Colossoma macropomum*) [69] and gilthead sea bream (*Sparus aurata*) [70].

### 4.4. Heat Shock Protein as Thermal Stress Biomarkers

The cellular response to thermal stress is a key process to preserve protein integrity; this is known as the heat shock response, which includes heat shock proteins (Hsps) to re-fold the proteins damaged by temperature [71]. This response was observed in the liver of red cusk-eel with the enrichment of the term response to heat (GO:0009408), highlighting several heat shock proteins differentially expressed in response to high-temperature stress, including *hsp40*, *hsp60*, *hsp70*, *hsp90*, and *serpinh1*. The up-regulation of these Hsps was previously observed in other fish under thermal stress, with an increase in the liver of three-spined stickleback (*Gasterosteus aculeatus*) (*hsp60*, *hsp70*, and *hsp90*) [72], Atlantic salmon (*Salmo salar*) (*hsp70*) [73,74], Atlantic cod (*Gadus morhua*) (*hsp70* and *hsp90*) [74], and Wuchang bream (*Megalobrama amblycephala*) (*hsp60*, *hsp70,* and *hsp90*) [75]. The *hsp60* and *hsp70* expression levels were also regulated under thermal stress in the postovulatory eggs and skeletal muscle of red cusk-eel [23,24], showing that these genes could be valuable biomarkers of thermal stress in this species. However, the most up-regulated gene in the liver under high-temperature stress was *serpinh1* (5.7log_2_ Fold Change, Table S2). *SerpinH1* (also known as *Hsp47*) is a chaperone involved in the biosynthesis of collagen at the ER [76], with a key role in the restoration of homeostasis during high-temperature stress and oxidative stress [22]. It was shown to be a good biomarker for thermal stress in several salmonid species, including rainbow trout (*Oncorhynchus mykiss*), sockeye salmon (*Oncorhynchus nerka*), and Chinook salmon (*Oncorhynchus tshawytscha*) [3,77], as well as zebrafish [78], evidencing that *sherpinh1* is one of the most suitable transcriptional biomarkers of high-temperature stress in fish. This was also the case for red cusk-eel in this study, representing a useful tool to evaluate thermal stress status in this species under a climate change scenario.

## 5. Conclusions

The present study evaluated for the first time the effects of high-temperature stress in the liver of *G. chilensis*, using a multiple approach of plasmatic hepatic enzymes, oxidative damage evaluation and RNA-seq analysis. We showed that high-temperature stress under heatwaves ENSO-associated scenario generated a major effect in the liver, affecting hepatic enzymes, generating oxidative damage in this tissue, as well as generating an unfolded protein response at the molecular level in several associated pathways, including a heat shock response, evidencing the affection of red cusk-eel under this type of stressor. This study contributes to knowledge about thermal stress under a climate change scenario, generating candidate biomarkers for thermal stress evaluation in this species, information that should be relevant for the aquaculture and fisheries industry of red cusk-eel.

## Figures and Tables

**Table 1:** Primer sequence, amplicon size, Tm and efficiency of validated genes by qPCR.

Abbreviation	Gene Name	Forward Sequence	Reverse Sequence	Amplicon Size (bp)	Tm	Efficiency (%)
*taf12*	Transcription initiation factor TFIID subunit 12	GATCTGTAACGACGACGAAGAA	CAAATCAGAGGGACGTCATGTA	92	62	101
*actb*	Beta actin	TGTCCCTGTATGCTTCTGGT	CCCCTCTCAGTCAGAATCTTCAT	172	62	104
*hsp60*	Heat shock protein 60	GACGGTTCCAATCTCTACATCTC	CGCTCTCCAAACCAGTTACA	86	62	99
*hsp70*	Heat shock protein 70	AAGATCAGCGACGACGATAAG	CTGGTGCTCATACTCCTCTTTC	105	62	95
*ddit4*	DNA damage-inducible transcript 4	GGGAATGAGGAGTTTGGTACAT	GAAGGAAGTGGTGGACCTTATT	88	62	96
*gpx7*	Glutathione peroxidase 7-like	TCTCCTTCCCTCTGTTCAGTAA	GAAATTCCAGTCGGGCTCTT	104	62	99
*leptin*	Leptin	CGAAGAGACTTCCTGCTTCAC	CTGATGATCTGGGTGGACTTTC	110	62	104
*msh2*	DNA mismatch repair Msh2-like	GCCCGTTCCCAGATATTTGAT	CGACCGCAATGACTACTACAC	100	62	98
*msh3*	DNA mismatch repair Msh3-like	CGACTTCTTCAGGGACTTTGG	TGGCTCTCTGAGTGTCTGT	78	62	104
*clql1*	Complement C1q 2-like	GATGTTTGTGGCGACGTATTTG	GTTGCTTTCTCAGCCTCTGTA	99	62	104
*ccl20*	C-C motif chemokine 20-like	CAGCCGTGTGTTAGGGAATA	CAGTTGTCTCGTGTCTCTCTATC	123	62	98
*atg12*	Ubiquitin ATG12-like	GCCCTCACCAGATCAAGAAG	AGAGAGTCAGAGTGGAGTTAGAG	133	62	102
*atg4b*	Cysteine protease ATG4B-like	ATCTGGGCGATCTGATGAATG	CGGAGGGCAGAAACAAAGA	96	62	102
*casp3*	Caspase 3	GCTCCAATTCTTTCCCGTATTT	CAGATTTCCTCTACGCCTACTC	123	61	103
*c3*	Complement C3-like	CTGCTTCTGGTGACCTGTTTA	CTTCGTGTCCTCTCCATCTTTC	99	62	103

**Table 2:** Sequencing and mapping statistics of liver libraries from control and thermal stress groups of *G. chilensis*.

Experimental Samples	Number of Reads	Average Length Number of Reads	Number of Reads after Trimming	Average Length after Trimming	Percentage of Mapped Reads
Control 1	51,220,468	101	51,103,248	95.0	85.1
Control 2	43,384,328	101	43,371,547	94.9	86.0
Control 3	50,445,708	101	50,425,183	94.9	85.4
Control 4	70,505,074	101	70,462,858	94.9	84.7
Control 5	65,838,802	101	65,800,592	94.9	85.7
Control 6	57,706,004	101	57,677,646	94.9	85.3
Stress 1	61,733,308	101	61,711,175	94.9	86.8
Stress 2	50,630,806	101	50,612,518	94.9	84.1
Stress 3	58,086,704	101	58,053,699	94.9	84.5
Stress 4	65,071,756	101	65,045,365	94.9	85.5
Stress 5	62,370,664	101	62,343,212	94.8	84.0
Stress 6	59,632,644	101	59,610,421	94.9	85.8
**Average**	**58,052,189**	**101**	**58,018,122**	**94.9**	**85.2**

**Table 3:** Enriched GO terms represented in up-regulated transcripts in response to high-temperature stress in the liver of *G. chilensis*.

GO Name	GO Category	GO ID	FDR	N° of Transcripts
Protein folding	BP	GO:0006457	2.96 × 10^−11^	28
Establishment of protein localization	BP	GO:0045184	6.62 × 10^−3^	30
Protein transport	BP	GO:0015031	1.36 × 10^−2^	29
Nitrogen compound transport	BP	GO:0071705	1.65 × 10^−2^	33
Protein localization to endoplasmic reticulum	BP	GO:0070972	3.41 × 10^−2^	6
Macromolecule localization	BP	GO:0033036	3.41 × 10^−2^	36
Transport	BP	GO:0006810	3.41 × 10^−2^	96
Cellular protein localization	BP	GO:0034613	3.41 × 10^−2^	25
Organic substance transport	BP	GO:0071702	3.41 × 10^−2^	38
Protein localization	BP	GO:0008104	3.41 × 10^−2^	31
Response to heat	BP	GO:0009408	3.41 × 10^−2^	5
Cellular macromolecule localization	BP	GO:0070727	3.41 × 10^−2^	25
Establishment of localization	BP	GO:0051234	3.80 × 10^−2^	96
Golgi organization	BP	GO:0007030	3.80 × 10^−2^	4
Protein retention in ER lumen	BP	GO:0006621	4.09 × 10^−2^	3
Maintenance of protein localization in organelle	BP	GO:0072595	4.09 × 10^−2^	3
Maintenance of protein localization in endoplasmic reticulum	BP	GO:0035437	4.09 × 10^−2^	3
Intracellular transport	BP	GO:0046907	4.09 × 10^−2^	28
Endoplasmic reticulum	CC	GO:0005783	3.20 × 10^−6^	33
Cytoplasm	CC	GO:0005737	9.42 × 10^−6^	133
Nuclear outer membrane-endoplasmic reticulum membrane network	CC	GO:0042175	1.31 × 10^−5^	24
Endoplasmic reticulum sub-compartment	CC	GO:0098827	3.68 × 10^−5^	23
Endoplasmic reticulum membrane	CC	GO:0005789	3.68 × 10^−5^	23
Endomembrane system	CC	GO:0012505	6.46 × 10^−5^	50
Organelle sub-compartment	CC	GO:0031984	7.63 × 10^−4^	29
Organelle membrane	CC	GO:0031090	2.14 × 10^−3^	44
Sarcomere	CC	GO:0030017	3.00 × 10^−2^	7
Myofibril	CC	GO:0030016	3.00 × 10^−2^	7
Coated membrane	CC	GO:0048475	3.41 × 10^−2^	10
Membrane	CC	GO:0016020	3.41 × 10^−2^	225
Membrane coat	CC	GO:0030117	3.41 × 10^−2^	10
Coated vesicle membrane	CC	GO:0030662	3.41 × 10^−2^	7
Cytoplasmic vesicle membrane	CC	GO:0030659	3.41 × 10^−2^	8
Contractile fiber	CC	GO:0043292	3.41 × 10^−2^	7
Unfolded protein binding	MF	GO:0051082	1.67 × 10^−9^	19
Signal sequence binding	MF	GO:0005048	2.70 × 10^−3^	5
ER retention sequence binding	MF	GO:0046923	4.09 × 10^−2^	3
Phosphofructokinase activity	MF	GO:0008443	4.69 × 10^−2^	5

## Data Availability

The raw read sequences obtained from liver sequencing of red cusk-eel were deposited in NCBI under BioProject PRJNA835467, with BioSample accession number SAMN28102858, SAMN28102859, SAMN28102860, SAMN28102861, SAMN28102862, SAMN28102863, SAMN28102864, SAMN28102865, SAMN28102866, and SAMN28102867. The datasets generated and/or analyzed in the present study are available from the corresponding author upon reasonable request.

## Supplementary Materials

The following supporting information can be downloaded at: https://www.mdpi.com/article/10.3390/biology11070990/s1, Figure S1: Differentially expressed transcripts in response to high-temperature stress in *G. chilensis* expressed as average transcription per group; Figure S2: Differentially expressed transcripts and clustering groups in response to high-temperature stress in *G. chilensis*; Figure S3: Validation of selected differentially expressed transcripts by qPCR on liver of *G. chilensis* on response to high-temperature stress; Table S1: Down-regulated differentially expressed transcripts between control and stress groups of *G. chilensis*; Table S2: Up-regulated differentially expressed transcripts between control and stress groups of *G. chilensis*.
