# Supplementary material for: High-Temperature Stress Effect on the Red Cusk-Eel (Geypterus chilensis) Liver: Transcriptional Modulation and Oxidative Stress Damage
Source: Biology (Basel). 2022 Jun 29;11(7):990. doi: 10.3390/biology11070990 (PMC9312335; doi:10.3390/biology11070990)
Supplement: Supplementary file 1 [file biology-11-00990-s001.zip › biology-1743109-supplementary-final/Supplementary figures S1-S2-S3.pdf]

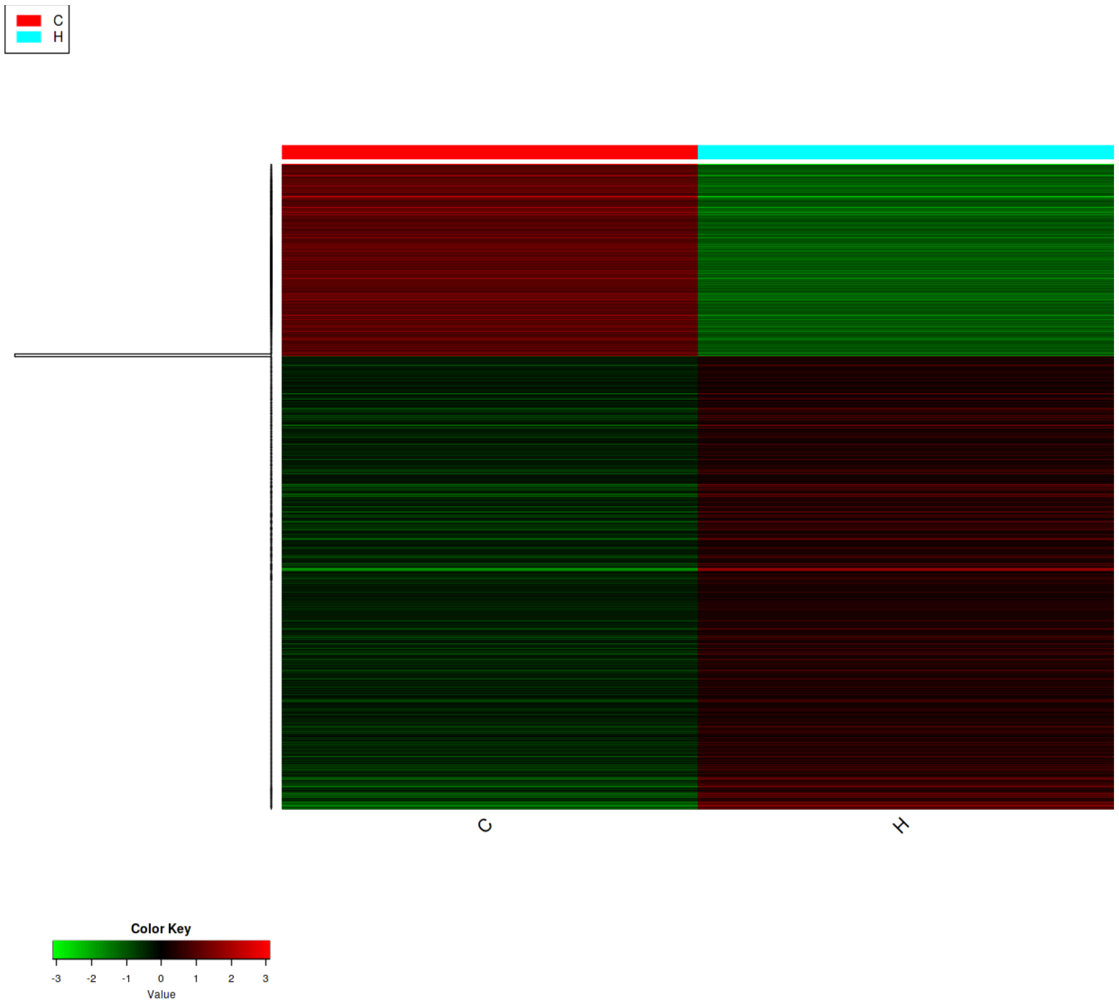

Figure S1. Differentially expressed transcripts in response to high-temperature stress in *G. chilensis* expressed as average transcription per group. The heatmap presents the pooled individual response of differentially expressed transcripts between control (C) and stress groups (H).

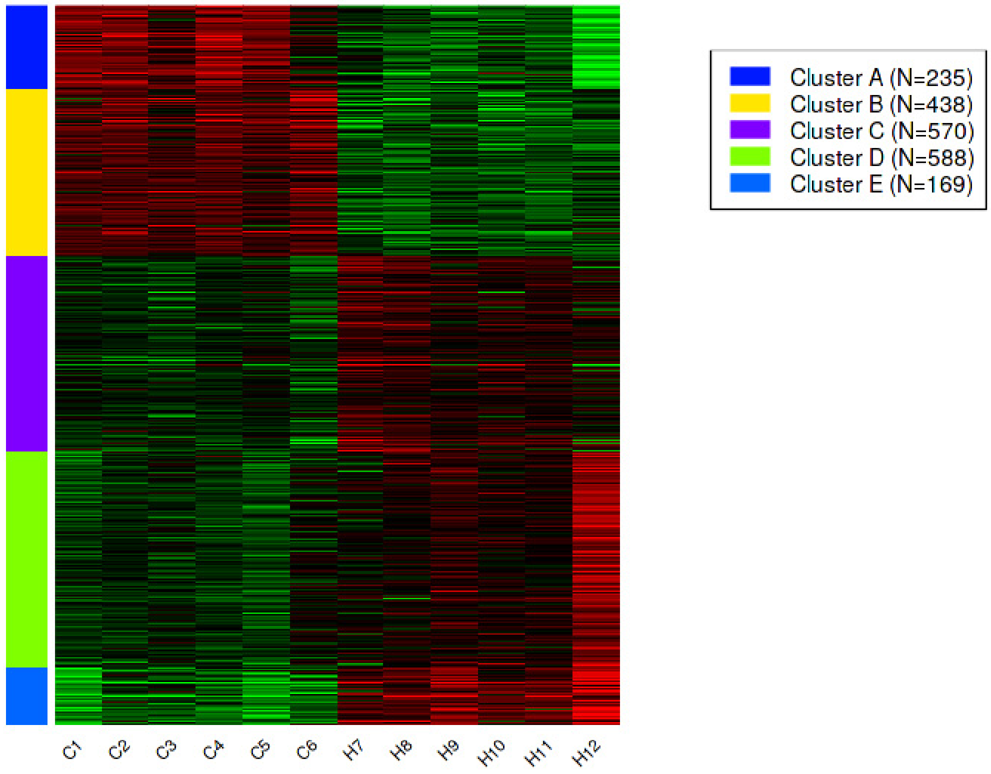

Figure S2. Differentially expressed transcripts and clustering groups in response to high-temperature stress in *G. chilensis*. The heatmap presents the pooled individual response of differentially expressed transcripts between control (C) and stress groups (H), as well as the clustering of transcripts with different patterns of expression for C and H groups. The number clusters (K) were determined according to Elbow method, considering the sum of squares.

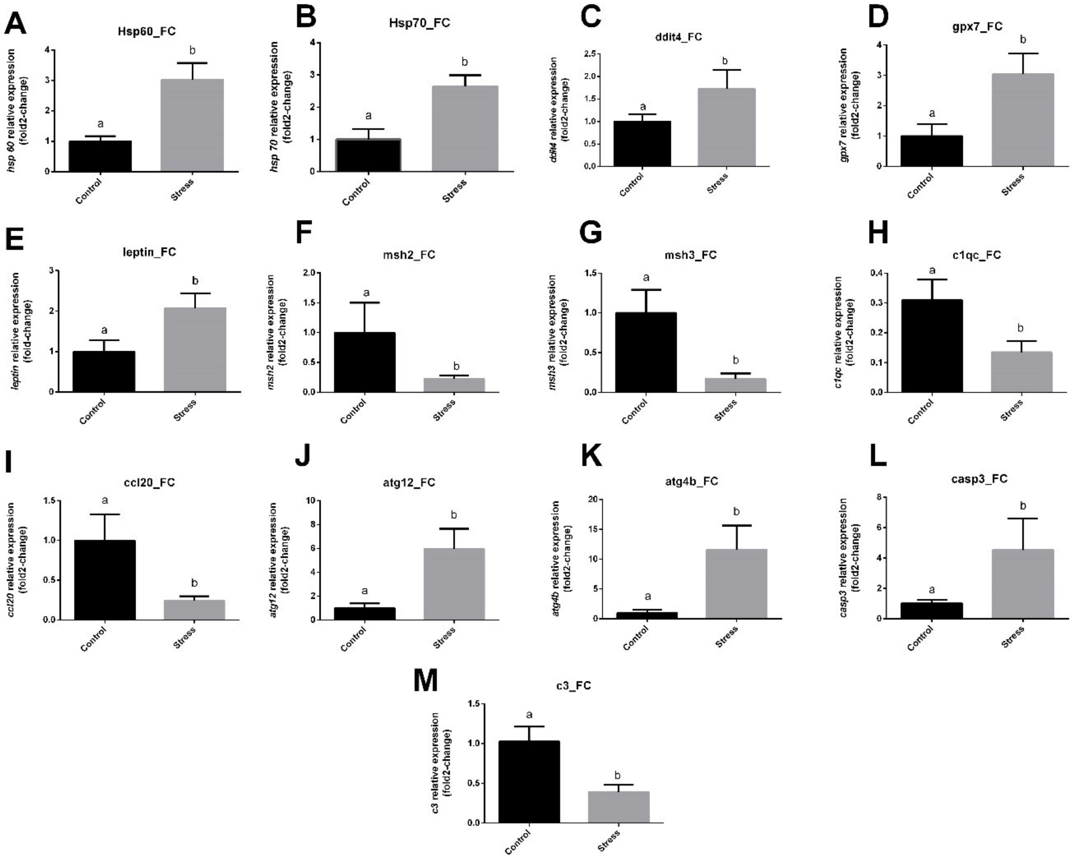

Figure S3. Validation of selected differentially expressed transcripts by qPCR on liver of G. c*hilensis* on response to high-temperature stress. The transcripts expression levels were normalized with the geometric means of *actb* and *taf12*. The relative expression of *hsp60* (A), *hsp70* (B), *ddit4* (C), *gpx7* (D), *leptin* (E), *msh2* (F), *msh3* (G), *clql1* (H), *ccl20* (I), *atg12* (J), *atg4b* (K), *casp3* (L), and *c3* (M) are expressed as log2 fold-changes for control and stress groups. Results are expressed as means ± standard error. Different letters indicate significant differences between groups (*p*-value < 0,05).
